# Feasibility of Achieving Nutritional Adequacy in Critically Ill Children with Critical Neurological Illnesses (CNIs)?—A Quaternary Hospital Experience

**DOI:** 10.3390/children11060711

**Published:** 2024-06-09

**Authors:** Marwa Mansour, Nicole Knebusch, Jennifer Daughtry, Thomas P. Fogarty, Fong Wilson Lam, Renan A. Orellana, Yi-Chen Lai, Jennifer Erklauer, Jorge A. Coss-Bu

**Affiliations:** 1 Division of Critical Care Medicine, Department of Pediatrics, Texas Children’s Hospital, Baylor College of Medicine, Houston, TX 77030, USA; msmansou@texaschildrens.org (M.M.);; 2 Department of Clinical Nutrition Services, Texas Children’s Hospital, Houston, TX 77030, USA; 3 Division of Pediatric Neurology and Developmental Neuroscience, Department of Pediatrics, Texas Children’s Hospital, Baylor College of Medicine, Houston, TX 77030, USA

**Keywords:** pediatrics, nutrition support, intensive care, critical neurological illness, malnutrition

## Abstract

The literature on the nutritional needs and outcomes of critically ill children is scarce, especially on those with critical neurological illnesses (CNIs). Current evidence shows a lower mortality in patients who achieve two-thirds of their nutritional needs during the first week of pediatric intensive care unit (PICU) admission. We hypothesized that achieving 60% of the recommended dietary intake during the first week of a PICU stay is not feasible in patients with CNI. We designed an observational retrospective cohort study where we included all index admissions to the PICU in our institution of children (1 month to 18 years) with CNI from January 2018 to June 2021. We collected patient demographics, anthropometric measures, and caloric and protein intake (enteral and parenteral) information during the first week of PICU admission. Goal adequacy for calories and protein was defined as [(intake/recommended) × 100] ≥ 60%. A total of 1112 patients were included in the nutrition assessment, 12% of whom were underweight (weight for age z score < −2). Of this group, 180 met the criteria for nutrition support evaluation. On the third day of admission, 50% of the patients < 2 years achieved caloric and protein goal adequacy, compared to 25% of patients > 2 years, with *p*-values of 0.0003 and 0.0004, respectively. Among the underweight patients, 60% achieved both caloric and protein goal adequacy by day 3 vs. 30% of non-underweight patients with *p*-values of 0.0006 and 0.002, respectively. The results show that achieving 60% of the recommended dietary intake by days 5 and 7 of admission was feasible in more than half of the patients in this cohort. Additionally, children who were evaluated by a clinical dietician during the first 48 h of PICU admission reached higher nutrition adequacy.

## 1. Introduction

The literature on nutrition needs estimation and provision for critically ill children is scarce. In addition to the lack of evidence, there are logistic difficulties in measuring the basal metabolic rate using indirect calorimetry (IC) and nitrogen balance in this vulnerable population. Despite these challenges, the available evidence supports that achieving a higher percentage of caloric and protein needs during the first week of PICU admission is associated with favorable outcomes, i.e., reduced mortality and PICU length of stay [1,2,3]. In a prospective observational multi-center study, Mehta et al. showed that achieving two-thirds of nutritional prescription during the first week of admission is associated with lower mortality [3].

The guidelines for the provision and assessment of nutrition support therapy by the Society of Critical Care Medicine (SCCM) and the American Society for Parenteral and Enteral Nutrition (ASPEN) recommend that in the absence of IC, Schofield/Food Agriculture Organization/World Health Organization/and United Nations University equations may be used, without the addition of stress factors to estimate energy needs. The guidelines also recommend a protein intake target of at least 1.5 gm/kg/day, a detailed nutrition assessment in the first 48 h of PICU admission, and a target energy intake of two-thirds of the prescribed daily energy requirement by the end of the first week in a PICU [4]. Observational studies have shown that achieving these targets in critically ill children is challenging due to inconsistent measurements and other patient-related factors, e.g., gut malfunction, difficult nasogastric/jejunal tube insertion, interruption of enteral or parenteral nutrition due to procedures and/or lack of access [1,5,6,7,8].

Critically ill patients are not only exposed to inadequate nutrition provision during their acute illness but are also commonly admitted to the pediatric intensive care unit (PICU) with a pre-existing diagnosis of malnutrition, which affects up to 60% of these patients [9,10,11].

Patients with critical neurological illness (CNI) are at particularly high risk of malnutrition due to neurological injury-induced adrenergic and neuroendocrine events believed to lead to a hypermetabolic state, in addition to possible pre-existing morbidities in patients with acute or chronic neurological diseases [12,13]. Evidence on nutrition for this heterogeneous and complex population remains limited [12,14].

Nutrition researchers use nutritional adequacy to express the intake level of an essential nutrient with the nutrient requirement for adequate health, which is described as the percentage of recommended dietary allowance (intake divided by prescribed) [3,15,16,17].

We hypothesize that achieving 60% of the recommended dietary allowance during the first week of a PICU stay is not feasible in patients with CNI. Our study aimed to undertake a nutrition assessment for patients admitted to a PICU with CNI and to evaluate nutrition adequacy (enteral and parenteral) during the first week of PICU admission. Additionally, we evaluated the effect of age and nutritional status in achieving the target nutrition adequacy during the first week of PICU admission within this patient population.

## 2. Materials and Methods

Our investigation entailed a retrospective cohort analysis of the index admissions to the pediatric intensive care unit (PICU) in our institution. We included children (aged one month to 18 years) with critical neurological illness (CNI), defined as any critical neurological illness that necessitates close monitoring in a PICU, from January 2018 to June 2021. The Institutional Review Board at Baylor College of Medicine, Houston (IRB: H-39403, approved on 17 March 2021), secured approval for this study’s ethical considerations.

A comprehensive set of data points were collected from information documented in the electronic medical record (EMR), encompassing patients’ demographics, anthropometric measures [weight (kilograms), height/length (cm)] upon admission, length of PICU stay, length of hospital stay, and duration of mechanical ventilation (MV). On admission, the risk of mortality (ROM%) was calculated using the Pediatric Index of Mortality 2 (PIM2).

### 2.1. Nutrition Assessment

We used z scores derived from anthropometric measurements, aligning with the growth curves by the World Health Organization (WHO), for patients below two years of age, and utilized growth curves, according to the Centers for Disease Control and Prevention (CDC), for patients aged two and above [18]. Specifically, the anthropometric measures employed were weight-for-age (WFA), height-for-age (HFA) z scores, and body mass index-for-age (BMI/A) or weight-for-length (WFL) z scores. Underweight was defined as a weight-for-age z score of <−2.0, acute malnutrition was defined as a weight for height z score of <−2.0, chronic malnutrition was defined as a height for age z score of <−2.0, overweight was defined as a weight for height with a z score of >2.0, and obesity was defined as a weight for height z score of >3.0. All nutritional categories were based on age and gender.

### 2.2. Nutrition Support

A subset of the cohort met the criteria of (1) >7 days of PICU stay, and (2) patients received nutrition intake by feeding tube or parenteral route. Patients with missing data and/or those who received oral intake were excluded. For this group of patients, we collected data from the electronic medical record on nutritional variables, including macronutrient (calories and protein) prescription by the clinical dietitians and the medical team, actual daily caloric and protein delivery, route of delivery (enteral vs. parenteral), and timing of clinical dietitian evaluation.

By using the Schofield equation, the basal metabolic rate (BMR) was used to estimate the daily recommended caloric intake [19]. Goal protein intake was calculated as 1.5 gm/kg/day for all ages. We identified nutrition goal adequacy as 60% of the recommended daily caloric and protein intake and calculated it as [(intake/recommended) × 100].

The primary outcome measure was the percentage of patients achieving goal nutrition adequacy during the first week of admission in the population admitted to PICU with CNI. Our assessment focused on several secondary outcomes, i.e., mortality, the duration of mechanical ventilation, and both PICU and hospital length of stay (LOS).

Descriptive statistics were used for sample distributions of the PICU, patient characteristics, and nutrition and clinical outcomes. Categorical variables are reported as counts and percentages, and continuous variables are summarized by their medians and quartiles. Analysis employed the chi-square test for comparisons of categorical variables. For continuous variables, the Mann–Whitney test was utilized to compare two independent groups. Statistical significance was established a priori *p* < 0.05. Statistical analysis was performed with Stat View Version 5.0.1 (SAS Institute Inc., Cary, NC, USA).

## 3. Results

### 3.1. Nutritional Assessment

**Figure 1:**
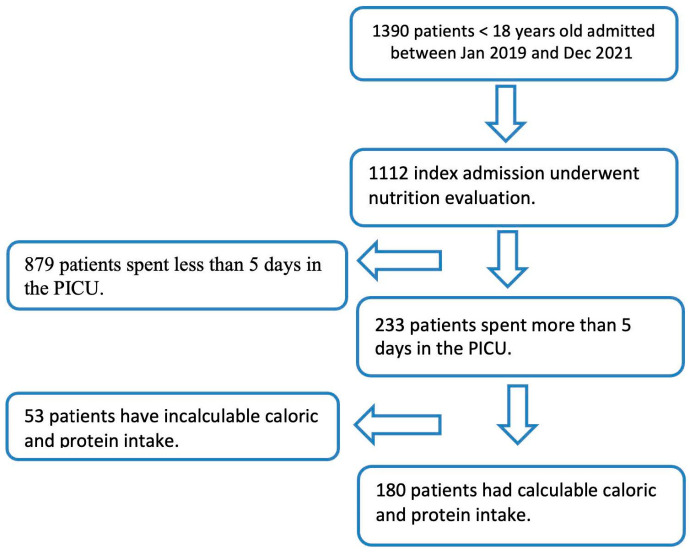
Flow diagram of patient inclusion in the study.

A total of 1112 patients were enrolled and included in the analysis, and 45% were females (Figure 1). The median and interquartile range (IQR) was 6.68 (1.94–12.9) years for age, 21.95 (11.7–45.4) kilograms for weight, ICU LOS 1.94 (1.05–4) days, hospital LOS 5.68 (3.47–15) days, MV duration 51 (20–153) h, and PIM2 ROM 1.03 (0.82–3.7)%. The prevalence of underweight was (12.14%, n = 135), and mortality was (1.70%, n = 19) (Table 1).

Diagnostic categories included seizures (35.5%), neurovascular (17.6%), infections (13%), neuromuscular (2%), spinal cord (3%), encephalopathy (11%), increased ICP (10%), extrapyramidal (3%), movement disorder (3%), neuro-degenerative disorder (2.5%), and trauma (3.5%).

We compared the clinical variables between underweight (n = 135, 12%) and non-underweight (n = 977, 88%) patients; age was 8.9 (1.0–13.8) vs. 6.6 (2.1–12.7) years, *p* = 0.8; PIM2 ROM 1.09 (0.83–3.75) vs. 1.03 (0.80–3.71)%, *p* = 0.5. The duration of MV in underweight patients (n = 54) was 98 (31–229) h vs. 47 (20–139) in non-underweight patients (n = 331), *p* = 0.006. ICU LOS was 2.9 (1.24–6.8) vs. 1.9 (1.0–3.9) days, *p* = 0.0008, and hospital LOS was 6.59 (3.7–17.64) vs. 5.6 (3.42–14.70) days, *p* = 0.13. Mortality was 3.70% (n = 5) vs. 1.43% (n = 14) (*p* = 0.056) (Table 2).

The comparison of clinical variables for patients < 2 years of age (n = 282, 25.4%) vs. >2 years of age (n = 830, 74.6%); PIM2 ROM 1.23 (0.92–4.13) vs. 0.99 (0.80–3.47) %, *p* < 0.0001. Duration of MV in patients < 2 years (n = 120) was 74 (28–225) h vs. in patients > 2 years (n = 265) it was 43 (18–115), *p* < 0.0005. ICU LOS was 2.6 (1.31–6.4) vs. 1.9 (1–3.7) days, *p* < 0.0001; hospital LOS was 7.2 (3.6–20.7) vs. 5.5 (3.44–13.71) days, *p* < 0.005. Underweight was 14.5% (n = 41) vs. 11.3% (n = 94), *p* = 0.15, and mortality was 2.84% (n = 8) vs. 1.33% (n = 11) (*p* = 0.09) (Table 3).

### 3.2. Nutrition Support

Of 1112 patients, 180 met the criteria, of which 39% were females. Seven patients were on a ketogenic diet. The median (IQR) for age was 4.8 (0.8–12) years, for weight it was 17 (8.8–35) kilograms, MV duration (n = 148) was 170 (98–358) h, PIM2 ROM 3.7 (1–7) %, ICU LOS 11 (7–20) days, and hospital LOS 23 (14.5–42) days. The prevalence of underweight patients was 21.6%, (n = 39). The mortality rate was 0.5% (n = 9) (Table 4).

**Figure 2:**
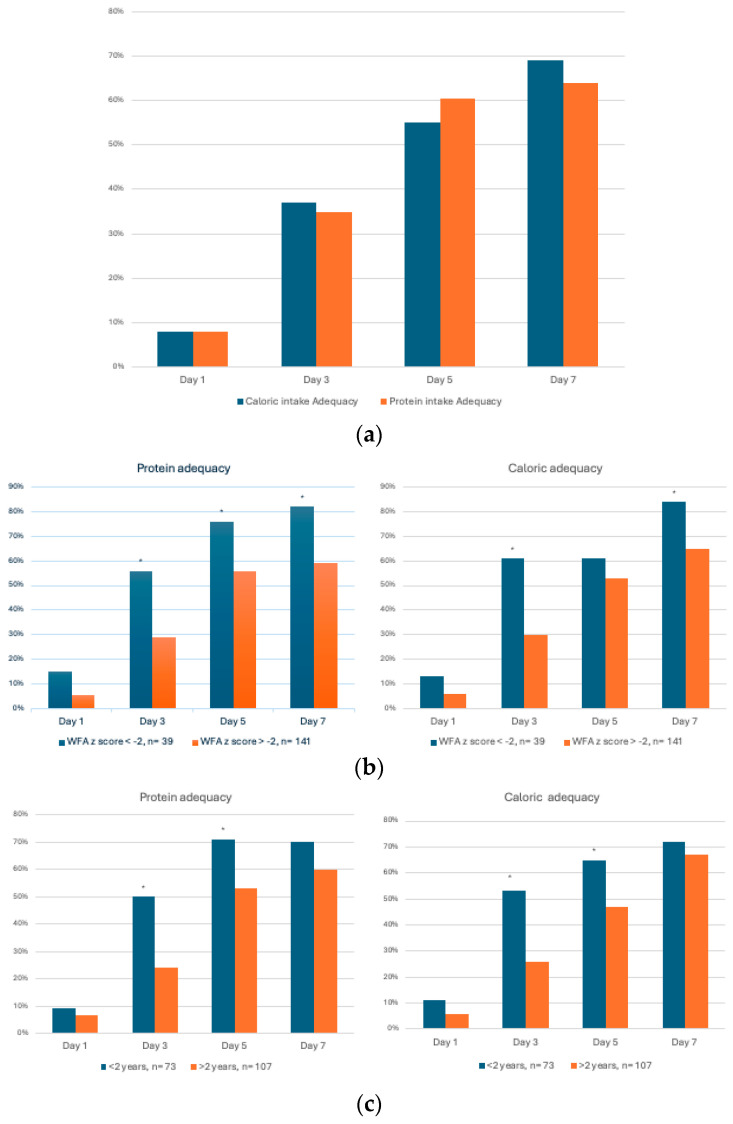
(**a**) Protein and caloric adequacy during the first week of admission in PICU. Values represent the percentage of patients who achieved protein and caloric goal adequacy (≥60%) during the first week of PICU admission. Adequacy: [(Intake/recommended) × 100]. (**b**) Protein and caloric adequacy by nutritional status. Values represent the percentage of patients who achieved protein and caloric goal adequacy (≥60%) during the first week of PICU admission. Comparison analysis for each day between groups; underweight defined as weight for age (WFA) z score < −2 vs. Non-underweight WFA z score > −2. * *p* value < 0.05 using chi-square test. (**c**) Protein and caloric adequacy by age. Values represent the percentage of patients who achieved protein and caloric goal adequacy (≥60%) during the first week of PICU admission. Comparison analysis for each day between groups; <2 years vs. >2 years. * *p* value < 0.05 using chi-square test.

The mean prescribed goal for calories was 58 ± 31 (SD) kcal/kg/day and 1.7 ± 0.6 g/kg/day for protein. Sixty percent of the patient population achieved the goal of nutritional adequacy for both calories and protein (delivered/recommended > 60%) at the end of the first week of PICU admission. The goal caloric intake adequacy was achieved on days 1, 3, 5, and 7 by 8% (n = 14), 37% (n = 67), 55% (n = 99), and 69% (n = 125) of the patients, respectively. The goal protein intake adequacy was achieved on days 1, 3, 5, and 7 by 8% (n = 14), 35% (n = 63), 60.5% (n = 109), and 64% (n = 116) of the patients, respectively (Figure 2a). There was no difference in mortality between patients who achieved nutrition adequacy and those who did not.

(a) Nutrition support in underweight vs. non-underweight patients

We compared achievement of optimal nutrition adequacy between underweight (n = 39) and non-underweight patients (n = 141).

Regarding caloric adequacy, 61% of the underweight patients achieved caloric adequacy by day 3 of admission versus 30% of the non-underweight patients, *p* = 0.0006. On days 5 and 7, 61% and 84% of the underweight patients achieved caloric adequacy compared to 53% and 65% of the non-underweight patients, *p* = 0.4 and 0.02, respectively. Regarding protein adequacy, 56% of the underweight patients achieved protein adequacy by day 3 of admission versus 29% of the non-underweight patients, *p* = 0.002. On days 5 and 7, 76% and 82% of underweight patients achieved protein adequacy compared to 56% and 59% of the non-underweight patients, *p* = 0.02 and 0.009, respectively (Figure 2b).

(b) Protein and caloric adequacy by age

We compared achievement of optimal nutritional adequacy between patients younger (n = 73) and older (n = 107) than two years of age.

Regarding energy adequacy, 53% of the patients under two years achieved optimal caloric adequacy by the 3rd day of admission, compared to 26% of the patients older than two years, *p* = 0.0003. On day 5, 65% of the patients < 2 years old achieved protein adequacy compared to 47% of the older patients, *p* = 0.02. Regarding protein adequacy, 50% of the patients under two years achieved optimal protein adequacy by the 3rd day of admission, compared to 24% of the patients older than two years, *p* = 0.0004. On day 5, 71% of the patients < 2 years old achieved protein adequacy compared to 53% of the older patients, *p* = 0.02 (Figure 2c).

Nighty-eight patients (55%) were evaluated and given recommendations by the clinical dietitian in the first 48 h. Patients who were evaluated by clinical dieticians during the first 48 h achieved higher nutritional adequacy compared to their counterparts on day 3 and day 7. There was no statistically significant difference in achieving goal caloric and protein adequacy (>60%) between these two groups. We compared the caloric and protein adequacies (%) between both groups, as shown in (Table 5).

There was no difference among various diagnostic neurological categories regarding achieving nutritional adequacy (Table 6).

## 4. Discussion

Pediatric malnutrition is an imbalance between the body’s nutrient needs and energy, protein, or micronutrient intake. This disequilibrium leads to cumulative deficiencies that can negatively impact growth, development, and other relevant outcomes [20]. Among critically ill children, the prevalence of malnutrition is reported to affect up to 60% of the patients worldwide [9,10,11].

Our study on patients with CNI in our institution showed that 12% of the patients were diagnosed as underweight, 22.5% exhibited signs of chronic malnutrition, and 8% were classified as acute malnutrition (Table 1). These results align with previously published reports of a ~20–30% prevalence of malnutrition in developed countries [21,22,23,24].

The literature establishes malnutrition as an independent risk factor for increased rates of infectious and non-infectious complications, higher mortality, prolonged hospital stays, and elevated healthcare costs [21,22,25]. In our cohort, underweight patients experienced prolonged mechanical ventilation and extended lengths of stay in the pediatric intensive care unit compared to non-underweight patients (Table 2).

These results align with the previously published literature demonstrating that underweight diagnosis is associated with longer intensive care length of stay and mechanical ventilation time, infections, and mortality [9,10,22,25].

Existing malnutrition is not the only factor that influences clinical outcomes for these patients; the critical illness itself may increase metabolic demand in the early stages of the stress response, and nutrient intake may be limited. Thus, children admitted to a PICU are at a higher risk of worsening nutritional status and anthropometric changes, both of which could correlate with adverse clinical outcomes [21,22,23,25,26].

Furthermore, patients with critical neurological illnesses are at risk of hypermetabolic and catabolic conditions, which are known to be associated with poor outcomes. Added to the critical neurological disease, poor nutritional status sets this vulnerable population at an increased risk of infections, more extended hospital stays, longer durations of mechanical ventilation, and higher mortality [5,13].

Hence, the American Society for Parenteral and Enteral Nutrition (ASPEN) and the Society of Critical Care Medicine (SCCM) have presented a comprehensive guideline for nutrition delivery in critically ill children, with significant emphasis on nutrition assessment—particularly the identification of malnourished patients, who are highly vulnerable and could benefit from prompt intervention. These guidelines suggest a standardized assortment of diagnostic markers for identifying and documenting pediatric malnutrition in routine clinical practice. The recommended indicators include z scores for weight for height/length, body mass index for age, length/height for age, or mid-upper arm circumference when a single data point is available [4,27].

These standardized diagnostic criteria permit precise nutritional assessment upon admission to a pediatric intensive care unit (PICU). This is pivotal in identifying children at risk for deteriorating nutrition, enabling interventions to optimize nutrient intake and potentially enhancing outcomes. In this study, we evaluated the nutritional status of patients admitted to a pediatric intensive care unit (PICU) with critical neurological illnesses to identify those with high-risk nutritional status and to evaluate the association between nutritional status and clinical outcomes.

Interestingly, although the malnutrition difference was not statistically different between age groups (>2 years and <2 years of age), we found that patients who were less than 2 years of age had higher pediatric mortality index scores upon admission. Also, they experienced longer mechanical ventilation times, longer ICU lengths of stay, and longer hospital lengths of stay (Table 3). These results warrant multi-center studies that compare clinical outcomes in relation to age in PICUs.

Regarding nutrition provision, the literature shows that early initiation and reaching at least two-thirds of the caloric and protein prescription in the general PICU population within the initial week of PICU admission are positively linked with shorter stays in the intensive care unit (ICU) and improved discharge statuses from the hospital [28,29].

Mehta et al. demonstrated that ensuring adequate caloric and protein intake during the first PICU week poses challenges. However, sufficient enteral protein intake was notably tied to reduced mortality among mechanically ventilated children [2,3].

The pediatric critical care population with critical neurological illness is diverse, and an individualized approach to nutrition support aiming to improve clinical outcomes is necessary, albeit with its inherent challenges. One of these challenges is that the body of literature that describes the caloric needs and optimal route of nutrition provision to pediatric patients with critical neurological illnesses is limited. Taha et al. showed that early enteral nutrition in patients with traumatic brain injuries (TBIs), a subset of patients with CNI, is associated with shorter intensive care length of stay [12]. These findings align with the 2019 TBI recommendations regarding an early initiation of enteral feeds in critically ill children, within the first 72 h when feasible.

Initiating early enteral nutritional support within this time frame is suggested to lower mortality rates and improve outcomes [14,30,31,32]. Balakrishnan et al. showed that patients with severe traumatic brain injuries were more prone to having delayed enteral nutrition initiation. They also proved that delayed enteral nutrition was independently associated with worse functional status at PICU discharge [33].

In our study, we focused on assessing the feasibility of achieving a goal nutrition adequacy of 60%. We looked into a subset of patients who spent more than 7 days in the PICU and received enteral (via enteral tube) and/or parenteral nutrition. A total of 180 patients met the criteria (Table 4). They had a higher Pediatric Index of Mortality than the rest of the cohort. The underweight prevalence of this cohort was 20%.

We observed that more than 50% of this cohort of patients with CNI achieved goal caloric and protein adequacy (goal intake > 60% of prescribed) by day 5 of admission, and >70% of the patients achieved that goal by the end of the first week of PICU admission (Figure 2a).

Over the same period, underweight patients consistently achieved the same goal of nutritional adequacy earlier and with higher intake than non-underweight patients (Figure 2b). Similarly, patients under two years old achieved nutritional adequacy earlier and with higher intakes to older patients (Figure 2c).

We observed that patients evaluated by the clinical dietician during the first 48 h were more likely to achieve higher caloric and protein intake during the first week of PICU admission compared to their counterparts (Table 5).

These results reflect what the other studies have demonstrated about the pivotal role of early assessment of critically ill patients by dieticians in pediatric ICUs [32,34,35].

We carried out a comparison of nutrition support across the different diagnostic neurological categories. However, the various diagnostic groups exhibited no variations in nutrition provision practices (Table 5).

This study was not powered to show a statistically significant difference in mortality between patients in this cohort who achieved nutrition adequacy and those who did not.

It is worth noting that the literature on the true caloric needs of patients with critical neurological illnesses is limited. Ideally, providers can utilize indirect calorimetry (IC) to determine critically ill patients’ basal metabolic needs. IC is a more accurate method than the commonly used equation, i.e., the World Health Organization (WHO) and Schofield equations, as they are proven to be inherently inaccurate in estimating the basal metabolic rate (BMR) of neurologically critically ill children [36,37].

However, due to technical difficulties and resource limitations, these equations are a more feasible method of estimating nutritional needs. In our study, we utilized the Schofield equation to calculate the BMR for each patient as the best available tool, despite its inherent limitations [19].

## 5. Conclusions

In this study, we evaluated the nutritional status of patients admitted to the Pediatric Intensive Care Unit (PICU) with critical neurological illnesses to identify those with high-risk nutritional status and to evaluate the association between nutritional status and clinical outcomes. We also looked into the feasibility of achieving nutritional adequacy of 60%, and into the association between achieving that goal and the nutritional status and age.

In summary, most patients admitted to our institution CNI achieved a goal caloric and protein adequacy of 60% during the first week of PICU admission. Underweight and younger patients < 2 years old were more likely to achieve nutritional adequacy than their counterparts. Similarly, patients whom the dieticians evaluated during the first two days of admission were more likely to achieve higher nutritional adequacy.

## 6. Limitations

This retrospective cohort study has inherent limitations that warrant consideration. It is a single-center study and may reflect nutritional support practices from our institution. A primary concern is the reliance on archived medical records as the primary data source. While rigorous efforts were undertaken to ensure the accuracy and reliability of the data through meticulous record reviews and validation procedures, the inherent nature of retrospective data collection introduces the possibility of information bias. This bias may arise from documentation errors, misclassification of exposures or outcomes, and variations in record-keeping practices across healthcare providers. Furthermore, the availability of certain clinical variables essential for comprehensive analysis was constrained by the content of the medical records, limiting our ability to account for all potential confounding factors. We acknowledge the need for future multi-center studies with larger sample sizes and more extensive data collection methodologies to address these limitations and provide a more robust evaluation of the associations elucidated in this study, thereby enhancing the depth and precision of our findings.

This study was also not able to detect statistically significant differences between groups (patients who achieved goal nutritional adequacy vs. not) within the nutrition support cohort in terms of clinical outcomes, e.g., mortality, PICU LOS, etc.

The findings in this study warrant larger observation outcome studies that look at clinical outcomes in relation to timing, route, and amount of nutrition provision. They also warrant randomized controlled trials that look into clinical outcomes in relation to underfeeding vs. overfeeding in critically ill children.

## Figures and Tables

**Table 1:** Patient characteristics and nutritional assessment.

n = 1112	Median (IQR)
Age (years)	6.7 (1.9–13)
Weight (kg)	22 (11.7–45.4)
Height (n = 1099) (cm)	115 (83–147)
Medical diagnosis n (%)	847 (76)
Surgical diagnosis n (%)	265 (24)
PIM2 ROM %	1 (0.82–3.7)
LOS MV (n = 385) (h)	51 (20–153)
PICU LOS (days)	2 (1–4)
Hospital LOS (days)	5.7 (3.5–15)
WFA z score	−0.17 ± 2 (SD)
Prevalence of underweight, n (%)	135 (12.14)
HFA z score	−0.9 ± 2 (SD)
Prevalence of chronic malnutrition, n (%)	250 (22.5)
WFL/BMI z score	−0.45 ± 1.8 (SD)
Prevalence of acute malnutrition, n (%)	88 (7.9)
Mortality, n (%)	19 (1.70)

Continuous variables are represented as medians with interquartile ranges (25th–75th). Categorical variables are expressed as numbers and percentages. Kg: kilograms; SD: standard deviation; PIM2: Pediatric Index of Mortality 2; ROM: risk of mortality; PICU LOS: pediatric intensive care unit length of stay; LOS MV: length of stay on mechanical ventilation; WFA: weight for age; HFA: height for length; WFL: weight for length; BMI: body mass index.

**Table 2:** Comparison of clinical variables by nutritional status—weight for age (WFA).

	WFA > −2 n = 977	WFA < −2 n = 135	*p* Value
Age (years)	6.6 (2–13)	8.9 (1–14)	0.84
Female gender n (%)	446 (45.6)	58 (43)	0.56
PIM2 ROM %	1.03 (0.80–3.71)	1.09 (0.83–3.75)	0.5
MV (h)	47 (20–139) n = 331	98 (31–229) n = 54	0.0055
PICU LOS (days)	1.9 (1.03–3.8)	2.9 (1.2–6.8)	0.0008
Hospital LOS (days)	5.6 (3.4–14.7)	6.6 (3.7–17.6)	0.12
Mortality, n (%)	n = 14 (1.4)	n = 5 (3.7)	0.056

Continuous variables are represented as medians with interquartile ranges (25th–75th) and compared using Mann–Whitney test. Categorical variables are expressed as numbers and percentages and compared using the chi-square test. Underweight is defined as weight for age (WFA) z score < −2. PIM2: Pediatric Index of Mortality 2; ROM: risk of mortality; LOS MV: length of stay on mechanical ventilation; PICU LOS: pediatric intensive care unit length of stay.

**Table 3:** Comparison of clinical variables by age.

	Age < 2 Years Old n = 282	Age > 2 Years Old n = 830	*p* Value
PIM2 ROM %	1.23 (0.92–4.13)	0.99 (0.80–3.47)	*p* < 0.0001
MV (h) (n = 385)	74 (28–225) (n = 120)	43 (18–115) (n = 265)	*p* < 0.0005
PICU LOS (days)	2.6 (1.31–6.4)	1.9 (1–3.7)	*p* < 0.0001
Hospital LOS (days)	7.2 (3.6–20.7)	5.5 (3.44–13.71)	*p* < 0.005
Underweight n (%)	41 (14.5)	94 (11.3)	*p* = 0.15
Mortality, n (%)	8 (2.84)	11 (1.33)	*p* = 0.09

Continuous variables are represented as medians with interquartile ranges (25th–75th) and compared using Mann–Whitney test. Categorical variables are expressed as numbers and percentages and compared using the chi-square test. Underweight is defined as weight for age (WFA) z score < −2. PIM2: Pediatric Index of Mortality 2; ROM: risk of mortality; LOS MV: length of stay on mechanical ventilation; PICU LOS: pediatric intensive care unit length of stay.

**Table 4:** Patient characteristics for the nutrition support cohort.

n= 180	Median (IQR)
Age (years)	4.8 (0.8–12)
Female, n (%)	70 (39)
Weight (kg)	17 (8.8–35)
Height (cm)	99 (69–136)
PIM2 ROM %	3.7 (1–7)
MV (n =148) (h)	170 (98–358)
ICU LOS (days)	11 (7–20)
Hospital LOS (days)	23 (14.5–42)
WFA z score	−0.59 ± 2.31 (SD)
Prevalence of underweight, n (%)	39 (21.6)
Patients on ketogenic diet, n (%)	7 (3.8)
Mortality, n (%)	9 (5)

Continuous variables are represented as medians with interquartile ranges (25th–75th). Categorical variables are expressed as numbers and percentages. Kilograms; SD: standard deviation; PIM2: Pediatric Index of Mortality 2; ROM: risk of mortality; LOS MV: length of stay on mechanical ventilation; PICU LOS: pediatric intensive care unit length of stay; WFA: weight for age.

**Table 5:** Comparison of nutritional adequacy between patients who were evaluated by a clinical dietician in the first 48 h after admission to the intensive care unit and those who were not.

		Evaluated before 48 h. n = 98	Evaluated after 48 h. n = 82	*p* Value
Day 3	Caloric adequacy (%)	51 (8–93)	21 (0–77)	0.03
	Protein adequacy (%)	45 (0–96)	15 (0–69)	0.02
Day 5	Caloric adequacy (%)	76 (11–125)	57 (7–109)	0.12
	Protein adequacy (%)	90 (44–134)	64 (16–116)	0.0495
Day 7	Caloric adequacy (%)	103 (56–152)	86 (26–116)	0.007
	Protein adequacy (%)	98 (48–136)	72 (25–105)	0.006

Continuous variables are represented as medians with interquartile ranges (25th–75th) and compared suing Mann–Whitney test. Nutritional Adequacy is calculated as [(intake/recommended) × 100] for calories and protein.

**Table 6:** Nutrition support adequacy according to diagnostic categories.

	Protein Adequacy ≥ 60%					Caloric Adequacy ≥ 60%				
	Infection n = 41	Seizure n = 57	Neurovascular n = 32	Other n = 50	*p* Value	Infection n = 41	Seizure n = 57	Neurovascular n = 32	Other n = 50	*p* Value
Day 1	9.7% (n = 4)	3.5% (n = 2)	3% (n = 1)	14% (n = 7)	0.14	9.5% (n = 4)	3.5% (n = 2)	3% (n = 1)	14% (n = 7)	0.2
Day 3	31% (n = 13)	35% (n = 20)	22% (n = 7)	46% (n = 23)	0.15	36.5% (n = 15)	38.5% (n = 22)	28% (n = 9)	42% (n = 21)	0.1
Day 5	56% (n = 23)	63% (n = 36)	56% (n = 18)	64% (n = 32)	0.8	51% (n = 21)	67% (n = 38)	50% (n = 16)	48% (n = 24)	0.16
Day 7	65% (n = 27)	61% (n = 35)	56% (n = 18)	72% (n = 36)	0.48	73% (n = 30)	64% (n = 37)	65% (n = 21)	74% (n = 37)	0.09

Categorical variables are expressed as numbers and percentages and compared by the chi-square test. Nutritional adequacy is calculated as [(intake/recommended) × 100] for calories and protein.

## Data Availability

The original contributions presented in the study are included in the article, further inquiries can be directed to the corresponding author due to privacy reason.
